# A Triangular-Matrix-Based Spectral Encoding Method for Broadband Filtering and Reconstruction-Based Spectral Measurement

**DOI:** 10.3390/s24041215

**Published:** 2024-02-14

**Authors:** Pinliang Yue, Xiaoxu Wang

**Affiliations:** 1 Changchun Institute of Optics, Fine Mechanics and Physics, Chinese Academy of Sciences, Changchun 130033, China; yuepinliang22@mails.ucas.ac.cn; 2 University of Chinese Academy of Sciences, Beijing 100049, China

**Keywords:** spectral encoding, spectral reconstruction, triangular matrix, spectral imaging, miniaturized spectrometer

## Abstract

Broadband filtering and reconstruction-based spectral measurement represent a hot technical route for miniaturized spectral measurement; the measurement encoding scheme has a great effect on the spectral reconstruction fidelity. The existing spectral encoding schemes are usually complex and hard to implement; thus, the applications are severely limited. Considering this, here, a simple spectral encoding method based on a triangular matrix is designed. The condition number of the proposed spectral encoding system is estimated and demonstrated to be relatively low theoretically; then, verification experiments are carried out, and the results show that the proposed encoding can work well under precise or unprecise encoding and measurement conditions; therefore, the proposed scheme is demonstrated to be an effective trade-off of the spectral encoding efficiency and implementation cost.

## 1. Introduction

Spectroscopy analysis and spectrometer instrument technology are fundamental technologies in studying the composition and structure of materials, and various spectrometers based on different principles have been developed and are widely used for detection and quantification. Spectral measurement based on broadband filtering and computational reconstruction (referred to as broadband filtering and reconstruction-based spectral measurement, BFRSM) is a new type of computational spectral measurement technology with the advantages of high optical throughput and compact structure, so it has become a major technical route in the field of miniaturized spectral measurement [1,2,3].

The basic principle of BFRSM is different from the common dispersion-based spectrometers. BFRSM uses broadband filtering devices to encode the incident spectrum, and each exposure of the detector acquires the spectrally encoded and mixed signal; then, the incident spectrum estimation can be obtained by inverting the spectral encoding and measurement processes, namely, spectral reconstruction. As spectral encoding can be realized through many micro or nano light-modulating devices, BFRSM is very suitable for spectroscopy miniaturization. Various types of miniaturized spectrometer and spectral imaging devices have been developed based on BFRSM, such as film array-based spectrometers [4,5,6], quantum dot spectrometers [7,8,9], nanowire spectrometers [10,11], photonic crystal spectrometers and spectral imagers [12,13,14], and many other types of spectrometers and spectral imagers [15,16,17,18,19,20]. Moreover, as the detector for BFRSM acquires broadband spectral energy for each exposure, the optical throughput can be much higher than that of conventional dispersion-based spectrometers, so it is also broadly studied in remote sensing [21,22].

The spectral reconstruction process is a typical inverse problem which is very sensitive to the input parameter noise; thus, spectral reconstruction fidelity is one of the key issues for BFRSM. Adding constraints to the spectral measurement encoding is an effective way to solve this problem, and some methods based on compress sensing and machine learning have been used to design the spectral encoding of the spectral filters. Compressed-sensing-based spectral encoding requires the spectral response matrix to obey the restricted isometric property, and the designed spectral encodings, like the Gaussian random matrix, are always quite complex and very hard to realize [15,23,24,25]. Machine learning methods have the advantages of high spectral encoding efficiency and much higher spectral reconstruction speed [15,26]; but the designed spectral encodings are also irregular and hard to accurately realize for almost all kinds of spectral filter devices. Thus, the complexity of the designed encoding schemes is a major bottleneck that limits the application of BFRSM. And this problem is especially serious for BFRSM-based spectral imaging systems, as the pixel-level irregular filter array devices are even harder to achieve.

Therefore, it is necessary to develop an easy-to-achieve spectral encoding method that can guarantee spectral encoding efficiency and reconstruction accuracy as well. In this paper, we propose a triangular-matrix-based spectral encoding scheme using long- and short-wavelength pass filtering as spectral encoders to guarantee spectral reconstruction accuracy. First, the condition number of the spectral encoding scheme is estimated theoretically to illustrate the feasibility; then, its error tolerance ability is discussed via numerical simulation. Finally, simple validation experiments are carried out to verify the effectiveness of the proposed method.

## 2. Basic Principle

### 2.1. Basic BFRSM Measurement Model

The spectral information could then be measured directly like the common dispersion-based spectrometers; that is, by first splitting light using a dispersive or diffractive optical element, followed by a direct measurement of each spatially dispersed spectral channel. Another approach is to acquire the spectral information indirectly, incorporating multiplexed and encoded spectral measurements like classic Fourier transformation spectroscopy [27]. This method allows the system to benefit from Fellgett’s multiplex advantage [28] and to achieve an obvious gain in optical throughput at the cost of post-processing. BFRSM is one of these indirect computational spectral measurement methods.

**Figure 1:**
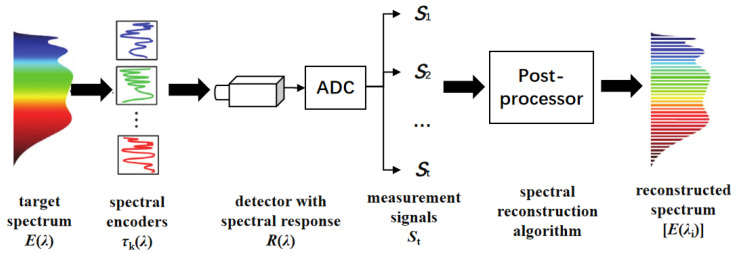
The principle of BFRSM technique.

Figure 1 shows the principle of broadband filtering-based spectral measurement. It can simply be understood as follows: the broadband light energy received by a photodetector can be regarded as the linear superposition of multiple narrowband lights corresponding to different central wavelengths; if multiple broadband filters are used as spectral encoders to encode the incident spectrum, and the spectrally encoded spectra are measured, respectively, for each encoding, then the discrete spectrum can be acquired by inverting the spectral measurement process shown in Equation (1). As this technique acquires broadband light instead of narrowband light, the optical throughput of one single exposure could be quite high. The output signal of the detector can be mathematically expressed as follows:

(1) $$S=\int_{\lambda_{0}}^{\lambda_{1}}R\left(\lambda \right)E\left(\lambda \right)d\lambda \approx \sum_{\lambda_{0}}^{\lambda_{1}}R\left(\lambda_{i}\right)E\left(\lambda_{i}\right),$$

where *S*, *R*(λ), and *E*(*λ*) are, respectively, the measured signal, the spectral response, and the spectral intensity of the discrete spectrum; *R*(*λ*_i_) and *E*(*λ*_i_) are, respectively, the discrete sampling of *R*(*λ*) and *E*(*λ*); *λ*_0_ and *λ*_i_ are, respectively, the lower and upper bound of the spectral response range of the system; and i represents the sequence number of the reconstructed spectral channels.

If the spectral response [*R*(*λ*_i_)] is encoded with different spectral filters with a spectral response of *τ*_*k*_(*λ*_i_) for *t* times (or the spectral response *R*(*λ*) is directly encoded for some specific devices), then Equation (1) can be written as follows:

(2) $$\left[S_{k}\right]=\left[\tau_{k}\left(\lambda_{i}\right)R\left(\lambda_{i}\right)\right]\left[E\left(\lambda_{i}\right)\right],$$

where [*S*_k_] is a column matrix with *t* × 1 elements, where each element of it represents the kth measurement signal corresponding to the *k*th spectral encoding; and [*E*(*λ*_i_)] is a column matrix, where each element of it represents the spectral intensity corresponding to the reconstructed spectral channel *λ*_i_.

It can be seen that a discrete spectral intensity [*E*(*λ*_i_)] can be acquired by solving Equation (2). Solving this problem is a typical inversion problem and it is sensitive to measurement noise. Restricting the observation matrix [*τ*_*k*_(*λ*_i_)*R*(*λ*_i_)] is effective for improving the inversion accuracy. 

### 2.2. Triangular-Matrix-Based Spectral Encoding

According to the linear algebra theory, the triangular matrix refers to the matrix whose non-zero coefficients are arranged like a triangle, and the triangular matrix has some special mathematical properties. One of these properties is that the triangular matrix has better well-posedness compared with other observation matrix forms; so, it is possible to design a spectral encoding method based on the triangular matrix.

**Figure 2:**
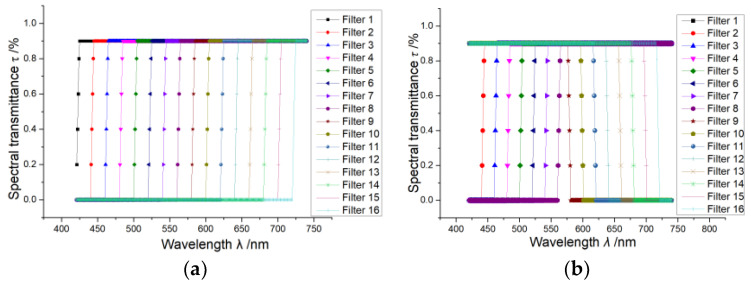
The designed spectral transmittance. (**a**) The long-wavelength pass spectral transmittance corresponding to the designed triangular-matrix-based spectral encoding matrix; (**b**) the modified spectral transmittance with high optical throughput.

The spectral observation matrix [*τ*_*k*_(*λ*_i_)*R*(*λ*_i_)] is the discrete sample of the continuous function *τ*_*k*_(*λ*)*R*(*λ*). If the spectral encoding filters are all long-wavelength pass filters with uniform-distributed cut-off wavelengths in the target spectral range, then the observation matrix [*τ*_*k*_(*λ*_i_)*R*(*λ*_i_)] would be of an approximate triangular matrix form. A transmittance numerical model is shown in Figure 2 to demonstrate this. The spectral transmittance of a designed long-wavelength pass filter set is shown in Figure 2a. It can be directly seen from the transmittance model that the detector can acquire broad spectral band energy in one exposure instead of narrowband spectral energy (compared with that of dispersion-based spectroscopy).

Here, when *τ*_*k*_(*λ*)*R*_*mn*_(*λ*) is uniformly sampled, *τ*_*k*_(*λ*)*R*_*mn*_(*λ*) can be expressed as follows:

(3) $$\left[\begin{array}{ccccc} a & 1 & \cdots & \cdots & 1\\ 0 & a & \ddots & \left(1\right) & \vdots \\ \vdots & \ddots & \ddots & \ddots & \vdots \\ \vdots & \left(0\right) & \ddots & \ddots & 1\\ 0 & \cdots & \cdots & 0 & a \end{array}\right]\circ \left[\begin{array}{ccccc} R(\lambda_{1}) & \cdots & R(\lambda_{i}) & \cdots & R(\lambda_{t})\\ R(\lambda_{1}) & \cdots & R(\lambda_{i}) & \cdots & R(\lambda_{t})\\ \vdots & \vdots & \vdots & \vdots & \vdots \\ \vdots & \vdots & \vdots & \vdots & \vdots \\ R(\lambda_{1}) & \cdots & R(\lambda_{i}) & \cdots & R(\lambda_{t}) \end{array}\right]=\left[\begin{array}{ccccc} aR(\lambda_{1}) & R(\lambda_{2}) & \cdots & \cdots & R(\lambda_{t})\\ 0 & aR(\lambda_{2}) & R(\lambda_{3}) & \cdots & R(\lambda_{t})\\ \vdots & \ddots & \ddots & \cdots & \vdots \\ \vdots & \left(0\right) & \ddots & \ddots & \vdots \\ 0 & \cdots & \cdots & \cdots & aR(\lambda_{t}) \end{array}\right]$$

where *α* is the sample value of the “rising edge” region of the designed spectral transmittance; *R*(*λ*_*i*_) is the discrete sample of *R*_*mn*_(*λ*); and ° represents the Hadamard product. It can be seen that the observation matrix [*R*_*mnk*_(*λ*_*i*_)] will be of a special triangular matrix form when long-wavelength pass filters with uniform cut-off wavelengths are used as spectral encoders.

It is worth noting that the total passband of the long-wavelength pass filters whose cut-off wavelengths are near the upper bound of the system spectral response range are quite narrow; hence, the optical throughput corresponding to these filters would be quite low. To solve this, the filters whose total passband is less than 50% of the entire spectral range can be replaced by short-wavelength pass filters with complementary spectral passbands, as shown in Figure 2b. In this way, all the measurements corresponding to each encoding filter can acquire at least 50% optical throughput of the entire spectral range. Under this condition, the observation matrix will be of a certain form, as follows.

(4) $$\left[\begin{array}{cccccc} 1 & 1 & \cdots & \cdots & \cdots & 1\\ \alpha & 1 & \cdots & \cdots & \cdots & 1\\ \vdots & \ddots & \vdots & \vdots & \vdots & \vdots \\ \left(0\right) & \cdots & \alpha & 1 & \cdots & 1\\ 1 & \cdots & 1 & 1-\alpha & \cdots & \left(0\right)\\ \vdots & \vdots & \vdots & \vdots & \ddots & \vdots \\ 1 & \cdots & \cdots & \cdots & 1 & 1-\alpha \end{array}\right]\to \left[\begin{array}{cccccc} 1 & 1 & \cdots & \cdots & \cdots & 1\\ \alpha & 1 & \cdots & \cdots & \cdots & 1\\ 0 & \ddots & \ddots & \vdots & \left(1\right) & \vdots \\ \vdots & \ddots & \alpha & 1 & \cdots & 1\\ \vdots & \cdots & \ddots & \ddots & \ddots & \vdots \\ \vdots & \left(0\right) & \vdots & \ddots & \ddots & 1\\ 0 & \cdots & \cdots & \cdots & 0 & \alpha \end{array}\right]$$

The two matrices shown in Equation (4) are not square matrix; elements in the first row are all 1, which represent the measurement without any spectral encoding. As can be seen, this kind of observation matrix can be transformed to the standard triangular form via simple elementary transformation. Therefore, the ill-posedness characteristic of the triangular matrix shown in Equation (3) can also represent that of modified longpass and shortpass filtering-based spectral encoding.

It is also worth mentioning that using a linear combination of Gaussian basis functions to approximate the reconstructed spectrum and sampling the spectral encoding is also a commonly used method, as illustrated in [11,24]. Using this method, the observation matrix [*R*_*mnk*_(*λ*_i_)] could also be transformed to a similar triangular matrix form, and its main ill-posedness property is also similar to that of the designed triangular matrix. 

### 2.3. Ill-Posedness Estimation

According to the algebraic theory of linear systems, the ill-posedness is the deterministic factor that affects the spectral reconstruction fidelity for solving Equation (2). As illustrated above, the observation matric shares a similar triangular form. Here, we use the *l*_1_ condition number to evaluate the ill-posedness of the proposed spectral encoding matrix (4), and only the positive definite matrix form is discussed here.

According to the definition, the condition number of [*R*_*mnk*_(*λ*_i_)] is as follows:

(5) $$cond=\left\|R_{\mathrm{mnk}}\left(\lambda_{i}\right)\right\|\cdot \left\|R_{mnk}^{-1}\left(\lambda_{i}\right)\right\|,$$

where *cond* is the condition number; $\left\|R_{\mathrm{mnk}}\left(\lambda_{i}\right)\right\|$ is the norm of [*R*_*mnk*_(*λ*_i_)]; and [*R*_*mnk*_(*λ*_i_)]^−1^ is the inverse of [*R*_*mnk*_(*λ*_i_)]. To simplify the evaluation, here, we adopt the *l*_1_ norm to evaluate the condition number.

According to the expression of spectral encoding matrix shown in Equation (3), the expression of its inverse matrix can be easily obtained:

(6) $$\left[\begin{array}{ccccc} \frac{1}{\alpha R_{1}} & \frac{-1}{\alpha^{2}R_{1}} & \frac{-\left(\alpha -1\right)}{\alpha^{3}R_{1}} & \cdots & \frac{-{\left(\alpha -1\right)}^{t-2}}{\alpha^{t}R_{1}}\\ 0 & \frac{1}{\alpha R_{2}} & \frac{-1}{\alpha^{2}R_{2}} & \cdots & \frac{-{\left(\alpha -1\right)}^{t-3}}{\alpha^{t-1}R_{2}}\\ \vdots & \ddots & \ddots & \frac{-{\left(\alpha -1\right)}^{j-i-1}}{\alpha^{j-i+1}R_{i}} & \cdots \\ \vdots & \left(0\right) & \ddots & \ddots & \cdots \\ 0 & \cdots & \cdots & 0 & \frac{1}{\alpha R_{t}} \end{array}\right]$$

where *R*(*λ*_i_) is written as *R*_i_ to simplify the expression; and *R*_i_ is the normalized relative spectral response of the system, which means 0 < *R*_i_ < 1. 

According to the expression of [*R*_*mnk*_(*λ*_i_)] and [*R*_*mnk*_(*λ*_i_)]^−1^, their *l*_1_ norm can, respectively, be derived as follows:

(7) $$\left\|R_{\mathrm{mnk}}\left(\lambda_{i}\right)\right\|=\alpha R_{1}+\sum_{2}^{t}R_{i},$$

(8) $$\left\|R_{mnk}^{-1}\left(\lambda_{i}\right)\right\|=\frac{1}{R_{p}}\cdot \frac{2k-{\left(\frac{1-k}{k}\right)}^{t-p}}{k\left(2k-1\right)},$$

where *p* is the row number corresponding to the specific row that has the maximum row sum.

Then, the *l*_1_ condition number *cond*_1_ can be acquired:

(9) $$cond_{1}=\left\|R_{\mathrm{mnk}}\left(\lambda_{i}\right)\right\|\cdot \left\|R_{mnk}^{-1}\left(\lambda_{i}\right)\right\|=\left(\frac{\alpha R_{1}+\sum_{2}^{t}R_{i}}{R_{p}}\right)\cdot \left[\frac{2\alpha -{\left(\frac{1-\alpha }{\alpha }\right)}^{t-p}}{\alpha \left(2\alpha -1\right)}\right].$$

Now we discuss the upper bound of *cond*_1_. As illustrated above, α is the sample value of the “rising edge” region of the spectral transmittance, and *R*_i_ denotes constants sampled from the spectral response; when measurement quantity *t* is a large number, the first multiplier in Equation (9) can be estimated as follows:

(10) $$\frac{\alpha R_{1}+\sum_{2}^{t}R_{i}}{R_{p}}\approx \frac{\alpha R_{1}}{R_{p}}+\frac{\int_{\lambda_{0}}^{\lambda_{1}}R\left(\lambda \right)d\lambda }{R_{p}\Delta \lambda }=\frac{\alpha R_{1}}{R_{p}}+\frac{\int_{\lambda_{0}}^{\lambda_{1}}R\left(\lambda \right)d\lambda }{R_{p}\left(\lambda_{1}-\lambda_{0}\right)}\cdot t,$$

where Δ*λ* is the total spectral response range; and *R*(*λ*) is the simplification of *R*_*mnk*_(*λ*). 

It can be seen in Equation (10) that for a specific system, $\int_{\lambda_{0}}^{\lambda_{1}}R\left(\lambda \right)d\lambda$, and other parameters are all constants that are determined by the spectral response property of the system; thus, $\frac{\alpha R_{1}+\sum_{2}^{t}R_{i}}{R_{p}}$ is approximately a linear function of measurement quantity *t*. For the second multiplier in Equation (10), if 0.5 < *α* < 1, ${\left(\frac{1-\alpha }{\alpha }\right)}^{t-p}$ decreases exponentially with *t*, and it tends to be $\frac{2}{2\alpha -1}$ when *t*→∞. If 0 < *α* < 0.5, ${\left(\frac{1-\alpha }{\alpha }\right)}^{t-p}$ is divergent when *t*→∞. 

It can be seen that when 0.5 < *α* < 1, the *l*_1_ condition number tends to be a parameter that increases linearly with *t* when *t* is pretty large. The constraint 0.5 < α < 1 can be simply understood as that at least 50% of the spectral bands in the “rising edge” region should be passbands; this is a relatively loose constraint for the encoding filters, which means a low implementation difficulty.

Therefore, the triangular-matrix-based spectral encoding scheme is not a completely orthogonal spectral encoding scheme. It is an ill-posedness-controllable system when the measurement system scale increases, which means a trade-off scheme between the measurement efficiency and the cost. It can work well in positive definite condition when the measurement scale is not very large. The spectral reconstruction algorithm for this encoding scheme is *l*_2_ norm minimization; it is sufficiently simple and rapid that it is suitable for spectral imaging application. 

## 3. Experimental Verification

### 3.1. Spectral Measurement under Precise Encoding Condition

An experiment facility was built to verify the performance of the proposed spectral encoding scheme. A Gershun radiometer composed of a well-calibrated silicon photodiode, a bandpass filter, and apertures was used as the detector. The bandpass filter was used to limit the spectral response range of the detector. A set of longpass filters placed in front of the radiometer were used as spectral encoding devices; changing the filters means changing different spectral encodings. An integrating sphere coupled with a supercontinuum fiber laser and a color filter was used as the target source; changing the color filter could change the source spectral distribution. 

A calibrated commercial spectrometer was used as the standard reference to verify the spectral reconstruction accuracy. The calibrated spectrometer consists of a CAS 140CT-152 compact array spectrometer and fiber-based input optics with a Gershun tube. The CAS 140CT-152 spectrometer (Instrument Systems GmbH, Munich, Germany) is a commercially available spectrometer whose working spectral range is 200–800 nm and whose spectral resolution is 2.7 nm. By changing the density filters integrated inside and adjusting the integration time of the CCD, the spectrometer can achieve a dynamic range of 10^9^. The light is guided into the spectrometer through an optical fiber. The other end of the optical fiber is naked and exposed; therefore, a Gershun tube is needed to restrict the field of view (FOV) of the spectrometer. The Gershun tube is mounted in front of the end of the optical fiber with a designed 5.6-degree FOV. We used a 1000 W FEL lamp provided by the National Institute of Standard and Technology (NIST), as well as a Labsphere Spectralon diffuser plaque with 8°/hemisphere reflectance data (calibrated by Labsphere) as a standard spectral radiance source, to calibrate the CAS spectrometer. After calibration, the CAS spectrometer with the Gershun tube aperture forms a standard spectroradiometer, with an absolute spectral radiance measurement uncertainty of about 2.7% at 555 nm. 

The detector is a high-accuracy Gershun radiometer, which consists of a NIST calibrated Si photodiode and an FOV aperture and can achieve about 0.3% measurement uncertainty in the spectral range of 400–800 nm. For more details about the Gershun radiometer and the reference commercial spectrometer, the reader may refer to [29].

**Figure 3:**
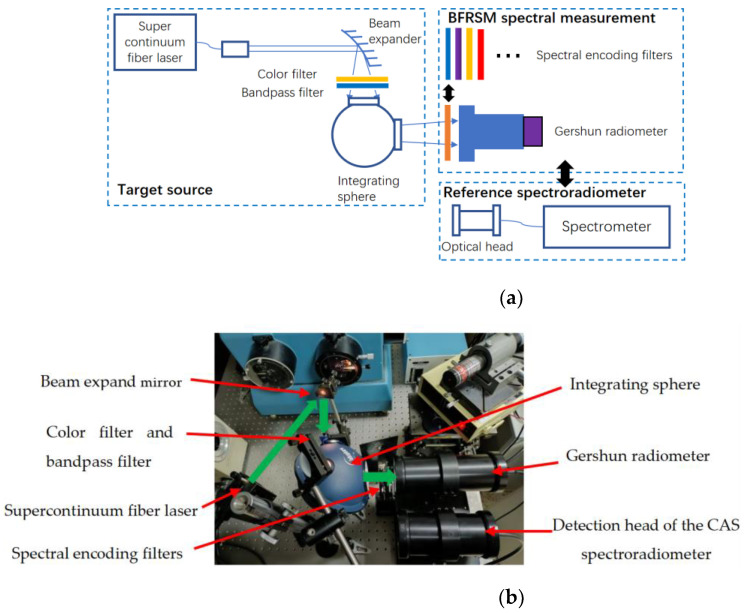
The encoding scheme verification experiment layout: (**a**) shows the schematic diagram of the experiment and (**b**) shows the actual experiment equipment.

The principle diagram of the experiment layout is shown in Figure 3a, and the actual experiment equipment is shown in Figure 3b. The green arrows represent the light path. The light emitted from the supercontinuum fiber laser (NKT photonics EXB-6, Birkerød, Denmark) was expanded and then passed through a color filter to modulate its spectral distribution, as the measurement reproducibility for various measurement targets needs to be verified. Another broad bandpass filter was placed after the color filter to limit the light spectral range; then, the light was guided into the integrating sphere. The spectral encoding filters were placed in front of the Gershun radiometer, and the light was encoded when passing through the spectral encoding color filters. The Gershun radiometer measured different encoded and mixed spectral signals as the encoding filters were changed; then, the signals and spectral transmittance of the encoding filters could be used to reconstruct the spectral radiance of the integration sphere. The reconstructed spectrum was verified by comparing it with the measured results of the CAS spectroradiometer.

**Figure 4:**
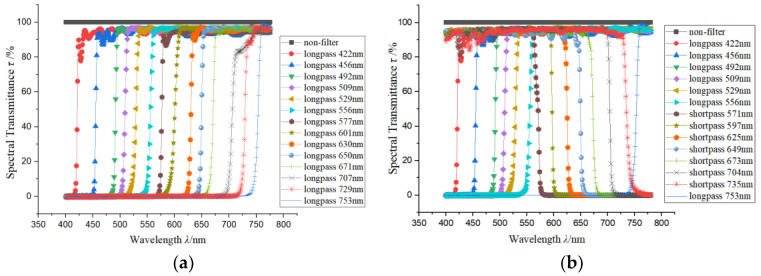
The spectral transmittance of the actual encoding filters: (**a**) shows the all-longpass-filter transmittance and (**b**) shows the modified spectral transmittance of the longpass and shortpass filters.

**Figure 5:**
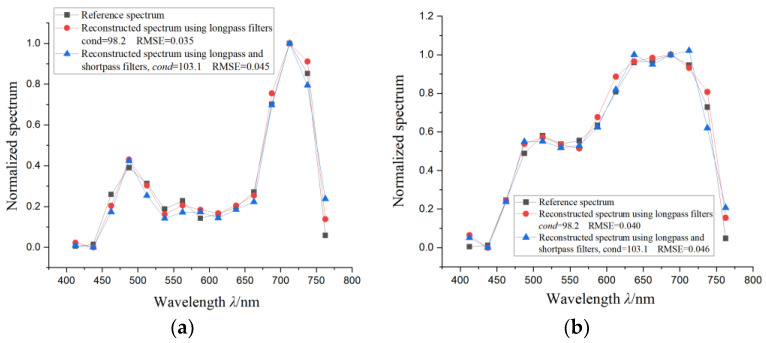
The comparison of the reference and reconstructed spectra: (**a**,**b**) shows two different reference spectra and the corresponding reconstructed spectrum using all-longpass filters and longpass–shortpass filters. The condition number and reconstruction RMSE are marked.

The spectral transmittance of the longpass filters used for spectral encoding is shown in Figure 4a. The spectral response range of the system was 400–775 nm, and every 25 nm was taken as one spectral channel, forming a total of 15 spectral channels. The discrete encoding values are all sampled simply from the spectral response using the average sampling method, and the spectral reconstruction algorithm was the basic non-negative least square algorithm without the regularization method. The quantity of the encoding filters and the reconstructed spectral channels are both 15. To demonstrate the reproducibility of the measurement results, we presented two different spectrum measurement results, shown in Figure 5a,b, consisting of the normalized reconstructed spectrum and the corresponding reference spectrum. The reference spectrum was also average-sampled from the original spectrum. The *l*_2_ norm condition number *cond* of the spectral encoding matrix and the spectral reconstruction RMSE are also marked. The measurement uncertainty of the experiment is shown in Table 1.

As can be seen, for the two different spectra, the proposed encoding method could provide pretty high spectral reconstruction fidelity and had similar spectral observation performances for different spectra. Using the simple non-negative least square algorithm for the proposed spectral encoding could provide enough spectral reconstruction fidelity, which indicates that the spectral image reconstruction speed will be high for spectral imaging application.

As illustrated above, changing the longpass filters with low synthetic transmittance to spectrally compensatory shortpass filters can increase the optical throughput, while it does not affect the reconstruction fidelity. To verify this, longpass and shortpass filters, shown in Figure 4b, are also used as spectral encoding devices. The replaced longpass filters in Figure 4a and the modified shortpass filters in Figure 4b have similar cut-off wavelengths. The reconstructed spectra using the longpass and shortpass filters are shown in Figure 5a,b. It can be seen that the spectral reconstruction RMSE using longpass and shortpass encoding is a little lower than that of the spectral reconstruction using the all-longpass filters. The reason is that the property of the used shortpass filters does not meet the encoding requirement very well, as their cut-off wavelengths are ununiformly distributed in the spectral range. But using longpass and shortpass filters as spectral encoding devices yields similar spectral measurement performances compared with only using the longpass filters, while the total optical efficiency is much higher, so this scheme is better for most applications. Additionally, Figure 5a,b show quite-close spectral reconstruction fidelities for two different target spectra using the same equipment; this means that the reproducibility of the method is pretty good.

In summary, the experiment results show that under pretty precise encoding and measurement conditions, the proposed spectral encoding method has pretty good spectral observation performance, and using longpass and shortpass filters with uniformly distributed cut-off wavelengths as spectral encoding devices yields similar spectral measurement performances while the optical throughput is much higher.

### 3.2. Spectral Measurement under Imperfect Encoding Condition

Generally, the spectral responses of the spectral encoding devices are not always ideal, and detectors like ordinary industrial cameras also have much greater noise than a single-pixel Si photodiode. Therefore, the proposed spectral encoding method should be tested under imprecise encoding and measurement conditions. Here, we used an industrial camera with pretty large noise as the detector and longpass color filters with a non-ideal triangular matrix encoding property as the spectral encoding device to verify the effectiveness of the proposed spectral encoding scheme under imprecise measurement conditions.

**Figure 6:**
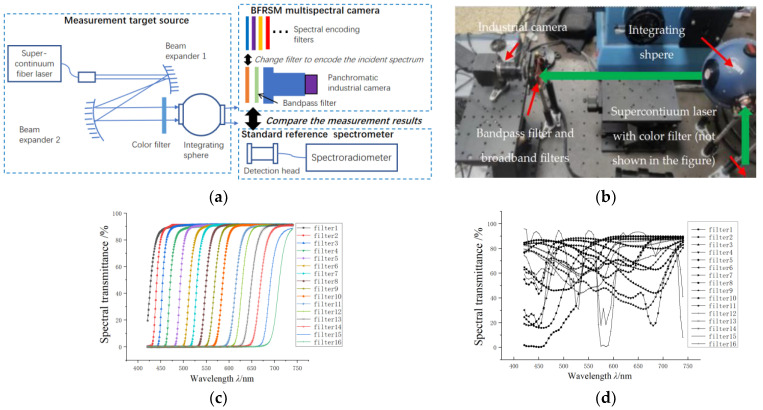
Experiment configuration principle and equipment for BFRSM spectral imaging: (**a**) shows the configuration and accuracy verification principle of the BFRSM multi-spectral camera; (**b**) shows the actual experiment equipment; (**c**) shows the longpass encoding filters with non-ideal spectral transmittance property; and (**d**) shows the 16 arbitrarily selected filters for spectral transmittance.

Figure 6a shows the configuration and the principle of the BFRSM multi-spectral camera: a panchromatic camera with calibrated spectral response was used as the detector, and a broad bandpass filter was placed in front of the camera to limit the spectral response range. The spectral response range of the BFRSM multi-spectral imaging system is 400–760 nm. A set of broadband filters are placed one after another in front of the camera system to encode the target spectrum. All the above formed a BFRSM multi-spectral imaging system. Figure 6b shows the actual spectral camera equipment.

Similar to the reference source shown in Figure 3, the reference target source in this experiment was also composed of a supercontinuum fiber laser with a spectral modulating color filter to change the source spectral distribution. Then, the light was guided into the integrating sphere, forming a uniform spectral source. 

Using the imaging system to image the target source, a set of spectrally encoded panchromatic images can be acquired. Then, the discrete spectrum can be reconstructed pixel by pixel, forming spectral images. The reconstructed spectrum was also compared with the standard reference spectrum measured via CAS spectroradiometer, illustrated above, to evaluate the spectral reconstruction accuracy. 

The spectral encoding filters are longpass color filters with gentle rising edges at the cut-off wavelengths. The rising edge region is all about 25 nm, which is much worse than that shown in Figure 4a (less than 4 nm). This represents a much worse spectral encoding accuracy for the designed triangular-matrix-based encoding and can partly represent the actual spectral encoding device manufacturing error. Figure 6c shows the spectral transmittance of the 16 non-ideal longpass encoding filters. 

Here, we used another 16 arbitrarily selected commercial color filters with different spectral transmittances as a control group to show the enhancement of the spectral encoding efficiency and spectral reconstruction fidelity using the proposed triangular-matrix-based encoding. Figure 6d shows the spectral encoding filters with arbitrary spectral transmittance.

**Figure 7:**
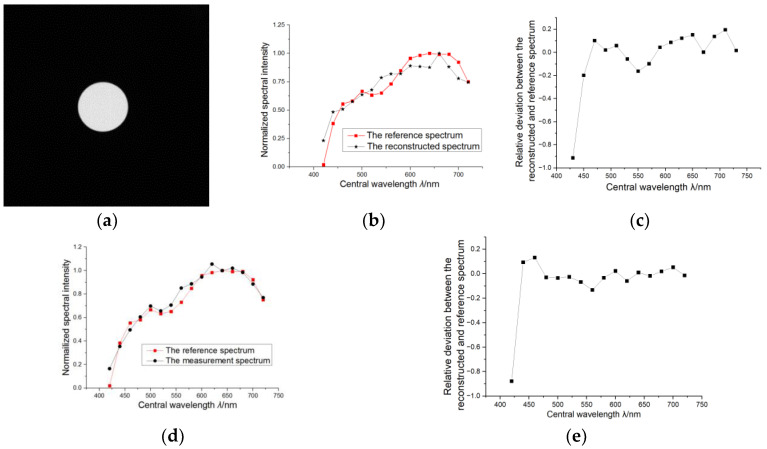
Spectral imaging experiment result: (**a**) shows the 550 nm spectral image of the target source; (**b**) shows the comparison between the reference and reconstructed spectra using arbitrary filter spectral encoding and (**c**) shows the relative deviation; (**d**) shows the comparison between the reference and reconstructed spectra using longpass filter spectral encoding and (**e**) shows the relative deviation.

Figure 7a shows the reconstructed 550 nm spectral image of the integrating sphere by the BFRSM multi-spectral camera, and the reconstructed spectra of 16 spectral channels using the arbitrary filters and the longpass filters are shown, respectively, in Figure 7b and Figure 7d; the relative error between the reconstructed spectrum and the standard reference spectrum is shown in Figure 7c,e. Here, the spectral reconstruction process used the generalized cross validation (GCV) Tikhonov regularization algorithm. The measurement uncertainty analysis of the BFRSM spectral imaging process is shown in Table 2.

In Figure 7a, it can be seen that the edge region of the reconstructed integrating sphere is clear, which shows that the spectral encoding and reconstruction process does npt introduce too much noise; and the non-uniformity of the bright region is about 6%, higher than the nominal 2% non-uniformity of the integrating sphere. It is caused by the camera noise and the spectral reconstruction process.

It can be seen that the actual deviation between the reconstructed and the reference spectra is about ±24%, and the relative RMSE is 0.118 (excluding the outlier of spectral band 1, its spectral intensity is nearly 0), which is much larger than the analyzed measurement uncertainty of the experiment process. In fact, the *l*_2_ norm condition number of the spectrum encoding matrix is 13,531; therefore, the inaccurate reconstruction result is theoretically predictable. And for the reconstructed spectrum corresponding to the longpass encoding filters, its relative error compared with the reference is about ±11%, and its relative RMSE is 0.067, which shows an obvious improvement compared with arbitrary spectral encoding. The *l*_2_ norm condition number of this non-ideal triangular encoding matrix is 569.

These results demonstrate that the proposed spectral encoding scheme is also effective when being used under non-ideal spectral encoding and imprecise measurement conditions. This indicates quite good tolerance for imperfect spectral encoding devices and shows a wide application potential for various types of spectral encoding devices.

## 4. Conclusions

The broadband filtering and computational reconstruction-based spectral measurement technique has the advantages of compact structure and high optical throughput; thus, it represents a hot technical route for the miniaturized spectrometer. To guarantee spectral observation efficiency and accuracy, special spectral observation matrix designs are necessary. The existing spectral encoding designs based on the orthogonal matrix, compressed sensing, or machine learning have good performance but are hard to implement. 

To balance the spectral observation efficiency and engineering difficulty, a triangular-matrix-property-based spectral encoding method is proposed in this paper. The *l*_1_ condition number of the typical proposed spectral encoding matrix is derived, and the ill-posedness of the proposed encoding system is discussed. Experiments were also carried out to verify the effectiveness of the proposed spectral encoding scheme. Theoretical analysis and experiment results show that the proposed spectral encoding method is effective under both precise and imprecise encoding and measurement conditions. 

But due to the limit of experiment’s resources and funds, the degradation of measurement accuracy with the increase in the encoding matrix scale has only been discussed theoretically; the performance of the proposed method under larger spectral encoding and measurement scales has not been well tested and evaluated. The linear growth of the ill-posedness may cause the complete failure of the proposed encoding scheme. But as the longpass and shortpass filters are quite easy to achieve for most spectral filtering devices, and as the corresponding spectral reconstruction algorithm is quite fast as its L2 norm minimization algorithm, we believe that the proposed method is suitable for the BFRSM spectral imaging system and BFRSM spectrometers with fewer spectral measurement scales and can provide some reference for the development of an easy-to-achieve spectral encoding method.

## Figures and Tables

**Table 1:** Uncertainty analysis of the precise spectral measurement experiment.

Uncertainty Source	Uncertainty Magnitude (k = 2)
Uniformity of the integrating sphere	1.0%
Uncertainty of the calibrated Gershun radiometer	0.5%
Stability of the laser source	0.8%
Uncertainty of the readout circuit	0.2%
Uncertainty of the spectral transmittance	1.2%
Calibration uncertainty of the reference spectrometer	3.5%
Combined uncertainty	4.0%

**Table 2:** Uncertainty analysis of the spectral imaging experiment.

Uncertainty Source	Uncertainty Magnitude (k = 2)
Uniformity of the integrating sphere	1.0%
Uncertainty of the industrial camera response	6.0%
Noise of the industrial camera signal (repeatability)	4.2%
Stability of the laser source	1.0%
Uncertainty of the readout circuit	0.2%
Uncertainty of the spectral transmittance	1.2%
Calibration uncertainty of the reference spectrometer	3.5%
Combined uncertainty	8.3%

## Data Availability

Data are contained within the article.
